# Ecosystem simulation: the software to platform leap

**DOI:** 10.1038/s41598-026-54641-7

**Published:** 2026-06-09

**Authors:** Zhe Liu, Yangjie Cui, Yudong Zhang, Zichen Li, Xiaoqing Liu, Shuihua Wang

**Affiliations:** 1 School of Management, Henan University of Technology, Zhengzhou, 450001 China; 2 School of Computer Science and Engineering, Southeast University, Nanjing, 211102 China; 3 Data Science and Computational Intelligence Institute, University of Granada, Granada, 52005 Spain; 4 Department of Biological Sciences, Xi’an Jiaotong-Liverpool University, Suzhou, 215123 China

**Keywords:** Industrial software, Platform transition, Ecosystem simulation, Tripartite evolutionary game, Engineering, Mathematics and computing

## Abstract

The transition from a software provider to a platform organizer is a pivotal transformation in the industrial software ecosystem (ISECO), enabling firms to fully leverage ecosystem dynamics, drive innovation, and achieve sustainable competitive advantages. Despite its growing significance, the strategic mechanisms underlying this transition remain underexplored. This study addresses this gap by employing an evolutionary game-theoretic approach to model the interplay among the government, the platform organizer, and the user in shaping ISECO’s trajectory across initial, mature, and ideal developmental stages. Through rigorous simulation analyses, we demonstrate that government subsidies and cost-sharing mechanisms significantly influence the evolutionary stability of the ecosystem. Furthermore, our findings reveal a critical threshold in cost-sharing strategies: a δ range of 0.2–0.4 that effectively incentivizes user adoption. When δ > 0.4, platform organizers may experience diminishing incentives to offer advanced services, potentially destabilizing the ISECO. This research advances the theoretical understanding of platform evolution by formalizing the dynamic, multi-agent interactions and threshold effects that drive this shift. It provides actionable insights for policymakers, software developers, and industrial strategists to design stage-specific subsidy withdrawal mechanisms and optimize cost-sharing structures without compromising profitability. The results underscore the necessity of dynamic, stage-specific interventions to facilitate a sustainable transition from software provision to platform organization.

## Introduction

In the digital transformation era, enterprises increasingly adopt platform strategies to create value and secure a competitive edge^1–3^. Unlike traditional pipeline models, platforms create and capture value by orchestrating networks of external producers and consumers, reducing transaction costs, and facilitating dynamic resource sharing^4^. These platform dynamics have reshaped industrial landscapes, particularly in industrial software, where firms are transitioning from standalone software providers to ecosystem orchestrators^5,6^. This transition is driven by the need to enhance interoperability, foster cross-sector innovation, and unlock new revenue streams through multi-sided markets. Industrial software is fundamental to modern industry, driving R&D advancements, optimizing production processes, enhancing management efficiency, and improving the performance of industrial equipment^7^. With the advent of Industry 4.0, manufacturing has evolved into intelligent factories and smart manufacturing, heightening demand for software that handles complex analytics, automates tasks, and integrates diverse systems^8^. Transitioning to a platform organizer is, therefore, an increasingly strategic move for industrial software enterprises. This shift allows businesses to leverage ecosystem effects to extend market reach, amplify value creation, and differentiate themselves in a rapidly evolving market^9,10^. Many software companies have already embraced this path, extending their technological base to include external business units, thereby reducing development and innovation costs through technology reuse and broadening their customer base^11,12^.

Unlike conventional software business models, platform-based ecosystems thrive on network effects and stakeholder interdependencies, making their evolution inherently complex^1,13^. To address this complexity, related work has explored mechanisms of digital platform evolution, highlighting the foundational role of external interventions in overcoming early-stage adoption barriers. Government interventions, particularly subsidies and regulatory policies, are pivotal in mitigating initial adoption barriers and fostering long-term ecosystem stability^3^. Additionally, cost-sharing mechanisms between platform organizers and users significantly influence participation incentives and the sustainability of ecosystem growth^4,14^. Recent evolutionary game studies further corroborate that equitable cost-sharing coefficients and benefit distribution are the critical conditions for breaking the early-stage participation deadlock and achieving stable synergetic development^15,16^. While these available studies have successfully laid the foundation by identifying the individual roles of subsidies and cost-sharing, they predominantly address these factors within the context of already established, mature platforms rather than during the transformative leap from software to platform.

However, the strategic processes required for software providers to effectively transition into platform organizers remain underexplored in the literature^17,18^. This gap is particularly pronounced in three dimensions: First, extant literature predominantly focuses on “born-as-platform” firms or mature ecosystems in developed markets^4^, offering limited guidance for traditional software providers navigating institutional and technological constraints. Second, research underestimates the socio-political dynamics shaping platform transitions in state-influenced economies^19^. Third, existing models assume static governance structures, neglecting how emerging-economy policies dynamically reshape ecosystem evolution^20^. Despite these recognized factors, their strategic interplay and long-term evolutionary implications remain insufficiently addressed in prior research.

This gap is particularly critical in emerging economies, where national strategies explicitly mandate platform‑centric industrial upgrades. However, the strategic processes governing this shift remain inadequately explored in the existing literature, necessitating a comprehensive theoretical framework that elucidates the critical determinants of this transformation. Motivated by rapid technological advancements and market dynamics, this study investigates how software providers evolve into platform organizers. This paper aims to explore the intricacies of this transition, providing insights into the challenges and opportunities software providers face as they align their business models with emerging market demands and technological trends.

To contextualize our framework, we apply our model to the empirical setting of the Chinese industrial software market. As the world’s largest manufacturing economy, China has historically faced significant challenges in industrial software development. Due to latecomer disadvantages in core technologies, critical software tools for product lifecycle management (PLM) and computer-aided manufacturing (CAM) remain primarily controlled by international firms, such as Siemens and Dassault Systèmes^18^. In response, the Chinese government launched initiatives such as “Made in China 2025” and the “Industrial Internet Innovation and Development Plan” to cultivate indigenous industrial software ecosystems, aiming to reduce dependency on foreign technologies and enhance industrial resilience^8,21,22^. These policies promote platform-centric strategies to aggregate resources, foster cross-sector collaboration, and accelerate innovation cycles, further underscoring the need to understand software-to-platform transitions in this context. Although China’s software industry developed later than its global counterparts and remains relatively less mature^23,24^, companies like CAXA have made significant strides. CAXA’s transformation from a traditional software provider to a platform organizer exemplifies this shift, as it builds an open ISECO integrating various stakeholders, including businesses, government entities, and research institutions. Driven by this empirical reality, our study offers specific theoretical and practical contributions: it introduces a comprehensive model analyzing the strategic transformation from software providers to platform organizers incorporating government, software suppliers, and platform users; identifies critical factors such as government subsidies and cost-reduction strategies; uncovers transition complexities and equilibrium stability through system dynamics modeling and simulations; and provides actionable recommendations for policymakers, developers, and platform users emphasizing strategic alignment and stakeholder engagement. The rest of the paper is organized as follows: Sect. 2 reviews literature on industrial software ecosystems, platform strategies, and business ecosystems; Sect. 3 details the assumptions and presents the tripartite evolutionary game model; Sect. 4 employs simulations to explore the transition process; and Sect. 5 summarizes key findings, limitations, and future research directions.

## Literature review

### Industrial software ecosystem.

The concept of the “software ecosystem” (SECO) has gained prominence over the past decade, highlighting the significance of collaboration and interconnectivity beyond organizational boundaries^25,26^. SECO involves interconnected business units, a shared market for software and services, and a common technical foundation that includes reference architectures, core assets, and standards. This ecosystem exchanges information, resources, and artifacts^27^.

**Fig. 1:**
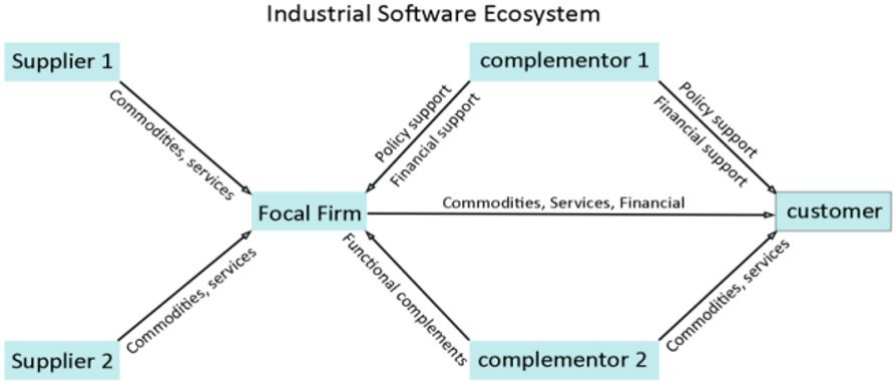
Elements of an ISECO.

The industrial software ecosystem (ISECO) is a dynamic network of interconnected software solutions, platforms, and actors that collaborate to drive innovation and efficiency in the industrial sector. As ecosystem theory gains traction, scholars are increasingly applying it across diverse fields, resulting in various ecosystem types, including business^28^, innovation^29,30^, platform^31,32^, and software ecosystems^33,34^. Originally developed to study natural environments^35^, the ecosystem concept has evolved to describe the intricate relationships between entities and their environments^35,36^, framing ecosystems as dynamic and interactive systems^37^. Contemporary definitions emphasize the collaboration among interconnected actors to enhance efficiency, create shared value, and shape the ecosystem’s trajectory^36,38,39^. Research on business ecosystems has revealed their complex network structures^40^, highlighting the interplay between value creation, value capture, and the balance between cooperation and competition. According to the models by Adner^31^ and Kapoor^32^, ISECO is a sophisticated network in which participants collaborate and compete to advance industrial software and services. Within this ecosystem, the focal firm, often the market leader, plays a crucial role by setting standards, guiding development, and creating value^41,42^. Suppliers contribute by offering advanced technologies and solutions that support the focal firm’s objectives and enhance the ecosystem’s overall competitiveness^43^. Complementors, such as those providing supplementary goods or services, including government policies, bolster the ecosystem’s cohesion. As key stakeholders, customers drive innovation and progress through their insights and feedback. For a detailed visualization, see Fig. 1.

### Platform strategy and digital transformation.

Digital transformation is the comprehensive integration of digital technologies into business operations, fundamentally reshaping how companies function and deliver value. Beyond adopting new technologies, strategic changes to business models, processes, and organizational culture are required to leverage digital opportunities^44^. Digital transformation is essential for growth, sustainability, and success in today’s rapidly changing market^45^. Serving as the strategic vehicle for this transformation, the platform strategy offers a promising approach to harnessing the power of modularity through a collaborative, dynamic ecosystem that drives growth and innovation^46,47^. Platform strategies represent a shift from a product-centric to an ecosystem-centric competitive model, in which value is co-created through modular architectures and networked complementarities^48^. Integrating digital technologies further strengthens the interconnectedness of the platform ecosystem, fostering closer collaboration among stakeholders and facilitating value co-creation^49–51^. In platform strategies, digital transformation accelerates ecosystem evolution by enhancing operational efficiency, driving innovation, and maintaining competitive advantage^52^. It also strengthens stakeholder collaboration, improves regulatory compliance, and enhances transparency and security through technologies such as blockchain^53^.

Driven by these digital shifts, in the field of industrial software, three strategic archetypes have emerged:

1. Integrated platforms, exemplified by Siemens Xcelerator, maintain tight control over core modules while curating third-party applications, ensuring high interoperability and security.
2. Open modular platforms, such as Eclipse IoT, minimize proprietary restrictions, allow third-party developers to contribute components, and foster a decentralized innovation model^48^.
3. Hybrid platforms where government-mandated standards govern the core layer, while application-level innovation remains market-driven^47^.

Each of these models presents distinct advantages and challenges. Integrated platforms offer reliability but can limit third-party contributions. Open modular platforms drive innovation but may struggle with governance and quality control. Hybrid platforms leverage regulatory advantages but require firms to navigate complex policy landscapes. Understanding these differences is crucial for firms designing platform strategies that align with technological trends and industrial policies.

Given these distinct platform typologies, enterprises must tailor their strategic approach to align with their chosen platform architecture. To successfully execute this strategy, enterprises must prioritize three key aspects: platform development, partner recruitment, and ecosystem construction. A robust technical architecture forms the foundation of platform development, which is essential for supporting current operations and for adaptability to future technological shifts^6^. An innovation-driven mindset keeps the enterprise at the forefront by exploring and integrating cutting-edge advancements^54^. An exceptional user experience, characterized by intuitive interfaces and seamless interactions, ensures high user satisfaction and loyalty^13^. After establishing a solid platform, focal firms can establish industry standards and forge strategic alliances with industry leaders^55^. To maintain strategic stability, focal firms must continuously enhance their platform’s functionalities, ensuring they align with evolving market demands and user expectations^56^. An incentive mechanism for developers and users fosters knowledge sharing, experience exchange, and problem-solving^57^. Additionally, collaborating with government agencies to secure policy support and resources creates a favorable external environment for platform growth^58^.

To gain a deeper understanding of these complex strategic implementation processes, some scholars have conducted comprehensive studies of platform dynamics, ecosystem formation, and the evolution trajectories of digital platforms. For instance, scholars have explored the dynamic governance mechanisms of network-driven ecosystems^59,60^, analyzed multi-agent co-innovation and cooperation strategies within established platforms using evolutionary game theory^61–63^, and revealed the inherent tensions and conflict resolutions during ecosystem evolution^64,65^. Furthermore, extensive research has been conducted on integrating broader business and social networks during digital transitions^66,67^, as well as evaluating the holistic impacts of digital transformation on enterprise innovation capabilities^68^. These cutting-edge works highlight that the success of a platform relies heavily on equitable multi-party interactions and continuous dynamic adaptation. However, integrating these recent insights reveals a critical research gap: while existing literature predominantly focuses on the governance, tension, and innovation dynamics within already mature or “born-digital” ecosystems, the specific evolutionary mechanisms underlying the initial, transformative “software-to-platform leap”—particularly how traditional software providers strategically transition into platform orchestrators—remain inadequately explored.

However, executing the transformative leap from a standalone software provider to a platform orchestrator is fraught with universal challenges. Globally, firms navigating this transition must overcome the “cold-start” problem inherent in multi-sided markets to bootstrap cross-side network effects and achieve early-stage critical mass^69,70^. Furthermore, they face the prohibitive R&D costs associated with building modular architectures and the complexity of designing governance mechanisms that align the conflicting incentives of diverse stakeholders. Traditional software providers often struggle to shift their organizational capabilities from product-centric innovation to ecosystem-centric orchestration, risking platform failure if these initial barriers are not overcome^71^.

These overarching structural and strategic challenges are particularly pronounced in emerging markets. When contextualized within the Chinese industrial software ecosystem, these universal barriers are amplified by latecomer disadvantages in core technologies and intense competition from established foreign incumbents. Consequently, Chinese firms cannot rely solely on organic, market-driven growth to complete the platform leap. Instead, navigating these amplified challenges requires a synergistic approach. Both endogenous challenges and exogenous policy incentives drive the adoption of platform strategies by Chinese industrial software firms:

Domestic drivers: The “dual circulation” economic strategy prioritizes the development of self-sufficient industrial chains, which necessitate platforms that bridge gaps among software developers, hardware suppliers, and manufacturers^22^. Additionally, fragmented domestic markets and high R&D costs incentivize platform adoption by enabling resource pooling and risk mitigation^18^. International drivers: Geopolitical tensions and supply chain disruptions have underscored the urgency of building resilient, localized ecosystems. Chinese firms are increasingly adopting platform-based models to reduce dependency on foreign software providers and enhance industrial autonomy^18^.

Platform strategies enable firms to capitalize on network effects, in which ecosystem value scales with increased participation by complementary actors^31^. Notable examples include Haier’s COSMOPlat, a platform integrating modular innovative manufacturing solutions that allows SMEs to access large-scale industrial resources, and Huawei’s MindSpore, which fosters AI-driven industrial applications through an open-source ecosystem^47^. These cases illustrate how Chinese industrial software firms leverage platform architectures to aggregate distributed innovation capabilities, transforming isolated software tools into interoperable solutions for smart factories.

## Business ecosystem and modeling techniques

The study of business ecosystems has advanced significantly in recent years, with scholars developing a range of modeling approaches to explore the complex interactions and dynamics within these systems. For instance, Agent-Based Modeling (ABM) captures complex, layered interactions and dynamic modeling^72^. However, ABM relies on predefined, often empirical behavioral rules. This highly parameterized, bottom-up nature makes it computationally opaque and difficult to achieve analytical tractability or derive strict mathematical equilibrium solutions^73,74^. System Dynamics (SD), while excellent for understanding overall ecosystem behavior and predicting long-term trends, may not fully capture the nuances of individual behavior^75^.

**Fig. 2:**
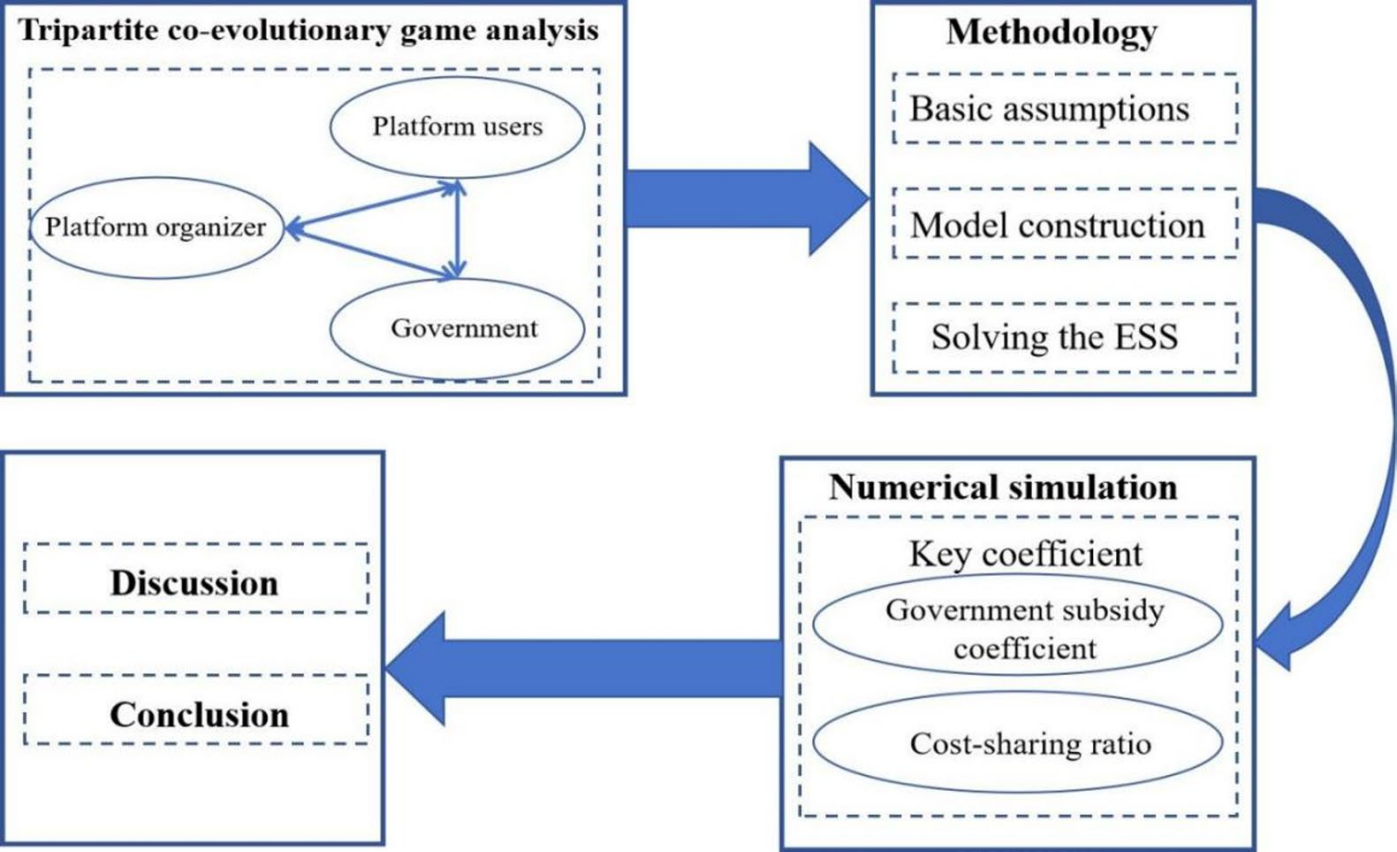
Framework of this research.

Evolutionary Game Theory (EGT) stands out for its ability to analyze interactions among multiple ecosystem stakeholders^24,76,77^. In contrast to ABM, EGT provides a robust analytical framework for evaluating the long-term strategic adjustments of boundedly rational populations. Through replicator dynamics and Jacobian matrix stability analysis, EGT allows for the rigorous mathematical deduction of exact macro-level threshold conditions (e.g., precise cost-sharing ratios and subsidy coefficients) necessary to reach an Evolutionarily Stable Strategy (ESS)^78^. This study uses EGT to simulate strategy evolution in business ecosystems, incorporating dynamic factors such as government subsidies and cost-sharing, and offers valuable insights for theoretical and policy applications. The study’s framework is illustrated in Fig. 2.

In the evolution of a business ecosystem, an enterprise often begins as a supplier in the nascent stages, adopting a niche strategy to carve out a specialized market space^79,80^. This approach allows the start-up to focus on specific customer needs that larger entities may overlook, thereby establishing a foothold and gaining a competitive edge in its chosen niche. As the enterprise matures, it accumulates capital and expertise, evolving from a niche player to an ecosystem creator. In this phase, the enterprise leverages platform strategies to build and manage digital technology platforms, fostering a network of suppliers, complementors, and partners that drive innovation and create shared value^5,79,81^. The ecosystem undergoes continuous restructuring, reflecting its dynamic nature and the necessity for adaptation and renewal^82^. With government support and policy incentives, the ecosystem leader can further enhance its stability and influence by welcoming external supplements that introduce new ideas and technologies^22^. It is valid for industrial software enterprises that innovation, research, and development have long been pivotal to maintaining competitiveness and achieving sustainable market growth^83^. However, faced with resource constraints, enterprises must reevaluate their ecosystem strategy to evolve and strengthen their position^84^. Strategic partnerships and technological alliances play a crucial role in this process, driving mutual benefits and enabling the exchange of knowledge and expertise^85^. This, in turn, generates fresh ideas and solutions that bridge technological gaps within the ecosystem, ultimately enhancing the overall experience for all users^86^.

## Problem description and assumptions

### Problem description

This study develops a three-party evolutionary game model to analyze strategic interactions among governments, platform organizers, and platform users in industrial software ecosystems. Unlike traditional software markets where interactions are mainly binary, such as supplier-user, platform ecosystems in emerging economies involve complex tripartite dynamics in which the government plays an active role as regulator, subsidy provider, and ecosystem enabler^13^.

Platform organizers are typically traditional software providers transitioning to a platform model, for whom strategic choices involve balancing investment in innovation and cost. Government subsidies may incentivize them to develop advanced platform features. However, they may also be tempted to misuse the funds by underinvesting in key features or failing to attract enough users due to poor ecosystem design^87^. This misallocation can lead to sub-optimal platform evolution or even ecosystem collapse without proper regulation.

For governments, the challenge is to design effective subsidy policies that stimulate platform adoption without creating long-term dependency. Thus, governments must carefully calibrate subsidies to ensure platform organizers invest in sustainable ecosystem growth rather than short-term profit maximization^88^. However, governments operate with imperfect information and cannot fully grasp how subsidies are used. This can lead to potentially risky situations in which platform organizers take advantage of financial support without delivering the expected innovation outcomes.

For platform users, the decision to adopt a platform depends on perceived benefits, such as access to advanced services and network effects, versus perceived risks, such as costs and platform instability. Users exhibit limited rationality, adjusting their strategies incrementally based on observed outcomes rather than making fully optimized decisions^89^.

**Fig. 3:**
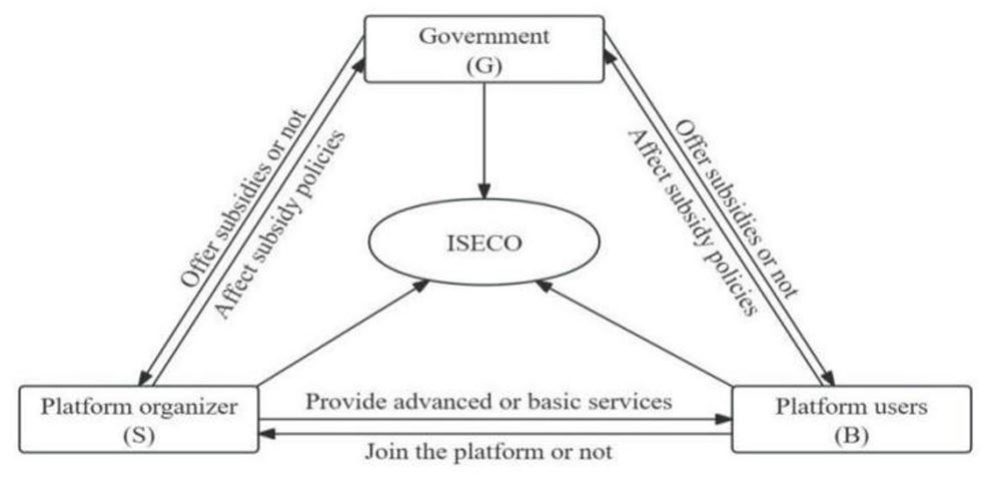
Relationships of stakeholders in ISECO.

In this simplified model of ISECO, a platform organizer makes key decisions about the level of service to offer—whether advanced or basic—and whether to share costs with users. Participants, in turn, evaluate the benefits and costs of joining the platform, influenced by the services offered and government subsidies, which aim to reduce their financial burden and encourage participation. The government plays a crucial role in shaping the platform’s dynamics through subsidy policies that must be both attractive and fiscally responsible. The interactions among these three entities form a complex game in which each player acts independently to maximize their own benefits. This leads to a dynamic system that seeks a stable balance in which all parties can thrive, ultimately driving the platform’s evolution. The game framework for the platform organizer, participants, and government is shown in Fig. 3.

Given these complexities, we employ an evolutionary game theory (EGT) framework to model how the three participants co-evolve their strategies. Unlike static game models, evolutionary game theory captures adaptive learning, whereby players iteratively improve their choices based on payoff comparisons. Our model specifically addresses the question of how government subsidy levels and regulatory interventions evolve as platforms mature. How does the platform organizer’s strategy shift from fundamental to advanced services? Under what conditions do users join the platform, and under what conditions do they remain independent?

### Model assumptions

The following hypotheses are formulated based on the intricate relationships among the tripartite entities depicted in Fig. 3.

#### Hypothesis 1

The strategic decisions of the platform organizer (*S*), platform users (*B*), and government (*G*) are mutually interdependent, leading to a dynamic equilibrium characterized by both competitive and cooperative tendencies. Specifically, the platform organizer has the option to provide advanced services, denoted by a probability *y* (0 ≤ *y* ≤ 1), or basic services, with a probability of (1 − *y*). Platform users can join the platform, represented by a probability x, or not, with probability (1 − *x*). The government, meanwhile, decides whether to provide subsidies, indicated by a probability *z*, or not to provide subsidies, with a probability of (1 − *z*). The intricate interdependencies and dynamic interactions among these strategic choices form the foundation of the game model, shaping its dynamics and equilibrium.

#### Hypothesis 2

When platform users abstain from joining the platform, their net profit is $\:{R}_{B}$. The decision to join the platform is intricately tied to the profit variance before and after joining, as well as the costs incurred. Furthermore, joining the platform exposes users to a heightened risk of losses when using its diverse functionalities. Specifically, the likelihood of suffering losses while utilizing advanced services is$\:\:{K}_{B}^{N}$, whereas for basic services, it is $\:{K}_{B}^{O}$. The maximum potential loss incurred by joining the platform is *Q*. To bolster user participation and foster the growth of ISECO, the government offers subsidies amounting to $\:\alpha\:{S}_{G}$ to users wherein $\:{S}_{G}$ represents the maximum subsidy allocated by the government and $\:\alpha\:$ serves as the subsidy allocation coefficient specific to platform users, constrained within the range of 0 ≤ $\:\alpha\:$ ≤ 1.

#### Hypothesis 3

Users who join the platform can access the platform organizer’s basic and advanced services. The profits accrued by the platform organizer and the users vary depending on the services used. Utilizing basic services yields direct benefits of $\:{R}_{B}^{O}$ for the users while generating profits of $\:{R}_{S}^{O}$ for the platform organizer. Conversely, when users choose to engage in advanced services, they stand to gain the direct benefits of $\:{R}_{B}^{N}$, and the platform organizer earns profits of $\:{R}_{S}^{N}$. Given that the platform organizer provides basic services at no cost, users must cover the necessary expenses, $\:{C}_{B}$ to unlock advanced services.

#### Hypothesis 4

To grow an ISECO, the platform organizer offers basic functions to users for free. This means the platform organizer bears the cost of providing these basic functions alone, denoted as $\:{C}_{S}^{O}$. To support the platform organizer, the government provides a subsidy of (1$\:-\alpha\:){S}_{G}$, where $\:{S}_{G}$ is the government’s maximum subsidy amount, and (1$\:-\alpha\:)$is the subsidy allocation coefficient for the platform organizer. In return for providing basic functions, the platform organizer gains additional benefits $\:{R}_{S}^{O}$, such as user usage data, user feedback, and user-developed plug-ins. To encourage users to select advanced services, the platform organizer shares a proportion $\:\delta\:$ of the cost $\:{C}_{B}$ for users. The revenue obtained by the platform organizer from providing advanced functions is $\:{R}_{S}^{N}$, and the cost incurred is $\:{C}_{S}^{N}$. As users become more reliant on these advanced functions, the platform organizer receives additional revenue $\:{I}_{S}$.

#### Hypothesis 5

ISECO can effectively drive advancements in industrial software and product innovation, enhancing digital transformation and boosting overall industrial competitiveness. When the government implements subsidy measures at a cost $\:{C}_{G}$, it yields significant social benefits $\:{F}_{B}$ (for subsidizing platform users) and $\:{F}_{S}$ (for supporting the platform organizer). As users join the platform, whether they utilize advanced or basic services, the government realizes revenue in the form of $\:{R}_{G}^{N}$ (for users of advanced functions) and $\:{R}_{G}^{O}$ (for users of basic functions).

For simplicity, we assume that when platform users opt out, the platform organizer incurs no revenue. However, despite the absence of user participation, the platform organizer decides to provide advanced services, and all government subsidies will be allocated solely to the platform organizer. The glossary of symbols is outlined in Table 1.

**Table 1:** Glossary of symbols and abbreviations.

Symbol	Description
*G*	Government
*z*	Probability of the government offering subsidies
1 − *z*	The probability of the government not offering subsidies
$\:{C}_{G}$	Cost of the government’s subsidy policies
$\:{R}_{G}^{N}$	Benefits to the government from advanced services
$\:{R}_{G}^{O}$	Benefits to the government from basic services
$\:{S}_{G}$	Total government subsidies allocated
$\:\alpha\:$	Government subsidy allocation coefficient, 0≤$\:\alpha\:$≤1
$\:{F}_{B}$	Social benefits from subsidizing users
$\:{F}_{S}$	Social benefits from subsidizing the platform organizer
*S*	Platform organizer
*y*	Probability of providing advanced services
1 − *y*	Probability of providing basic services
$\:{R}_{S}^{N}$	Benefits from users opting for advanced services
$\:{R}_{S}^{O}$	Benefits from users opting for basic services
$\:{C}_{S}^{N}$	Cost of providing advanced services
$\:{C}_{S}^{O}$	Cost of providing basic services
$\:{I}_{S}$	Additional benefits from increased user reliance on advanced services
$\:\delta\:$	Cost-sharing ratio with users, 0≤$\:\delta\:$≤1
*B*	Platform users
*x*	The probability of joining the platform
1 − *x*	The probability of not joining the platform
$\:{R}_{B}^{N}$	Benefits of using advanced services
$\:{R}_{B}^{O}$	Benefits of using basic services
$\:{C}_{B}$	Cost of using advanced services
$\:{K}_{B}^{N}$	Probability of incurring losses with advanced services
$\:{K}_{B}^{O}$	Probability of incurring losses with basic services
*Q*	Maximum loss from joining the platform
$\:{R}_{B}$	Net profit when not joining the platform

## Construction and analysis of evolutionary game models

### Payoff matrix

Based on the established assumptions, an evolutionary game model has been constructed, and the resulting payoff matrix is presented in Table 2. This matrix encapsulates the financial outcomes for each participant under various decision scenarios. It is a valuable tool for analyzing strategic choices and equilibrium outcomes within the game framework.

**Table 2:** Payoff matrix of tripartite.

Platform organizer	Government	Platform users	
		Joining the platform (*x*)	Not joining the platform (1 − *x*)
Providing advanced services (*y*)	Offering subsidy (*z*)	$$\:\left\{\begin{array}{c}{R}_{B}^{N}-(1-\alpha\:){C}_{B}-{K}_{B}^{N}Q+\alpha\:{S}_{G}\\\:{R}_{S}^{N}+(1-\alpha\:){S}_{G}-{C}_{S}^{N}+{I}_{S}-\delta\:{C}_{B}\\\:{R}_{G}^{N}-{C}_{G}-{S}_{G}+{F}_{S}+{F}_{B}\end{array}\right\}$$	$$\:\left\{\begin{array}{c}{R}_{B}\\\:-{C}_{S}^{N}+{S}_{G}\\\:-{C}_{G}-{S}_{G}+{F}_{S}\end{array}\right\}$$
	Not offering subsidy (1 − *z*)	$$\:\left\{\begin{array}{c}{R}_{B}^{N}-(1-\delta\:){C}_{B}-{K}_{B}^{N}Q\\\:{R}_{S}^{N}-{C}_{S}^{N}+{I}_{S}-\delta\:{C}_{B}\\\:{R}_{G}^{N}\end{array}\right\}$$	$$\:\left\{\begin{array}{c}{R}_{B}\\\:-{C}_{S}^{N}\\\:0\end{array}\right\}$$
Providing basic services (1 − *y*)	Offering subsidy (*z*)	$$\:\left\{\begin{array}{c}{R}_{B}^{O}+\alpha\:{S}_{G}-{K}_{B}^{O}Q\\\:{R}_{S}^{O}-{C}_{S}^{O}\\\:{R}_{G}^{O}-{C}_{G}-\alpha\:{S}_{G}+{F}_{B}\end{array}\right\}$$	$$\:\left\{\begin{array}{c}{R}_{B}\\\:-{C}_{S}^{O}\\\:-{C}_{G}\end{array}\right\}$$
	Not offering subsidy (1 − *z*)	$$\:\left\{\begin{array}{c}{R}_{B}^{O}-{K}_{B}^{O}Q\\\:{R}_{S}^{O}-{C}_{S}^{O}\\\:{R}_{G}^{O}\end{array}\right\}$$	$$\:\left\{\begin{array}{c}{R}_{B}\\\:-{C}_{S}^{O}\\\:0\end{array}\right\}$$

### Analysis of replication dynamics

#### Platform users

The expected payoff, $\:{\mathrm{E}}_{\mathrm{b}1}$, can be derived from Eq. (1) when platform users choose to join the platform.

1 $$\:{\mathrm{E}}_{\mathrm{b}1}=yz\left[{R}_{B}^{N}-\left(1-\delta\:\right){C}_{B}-{K}_{B}^{N}Q+\alpha\:{S}_{G}\right]+y\left(1-z\right)\left[{R}_{B}^{N}-\left(1-\delta\:\right){C}_{B}-\:\:\:\:\:\:\:{K}_{B}^{N}Q\right]+(1-y)z({R}_{B}^{O}+\alpha\:{S}_{G}-{K}_{B}^{O}Q)+(1-y)(1-z)({R}_{B}^{O}-{K}_{B}^{O}Q)$$

Otherwise, the expected payoff, $\:{\mathrm{E}}_{\mathrm{b}2}$, is specified in Eq. (2) when platform users choose not to join the platform.

2 $$\:{\mathrm{E}}_{\mathrm{b}2}=yz{R}_{B}+y(1-z){R}_{B}+(1-y)z{R}_{B}+(1-y)(1-z){R}_{B}$$

Thus, the average return, denoted as$\:{\mathrm{E}}_{\mathrm{b}}\:$, is calculated as follows.

3 $$\:{\mathrm{E}}_{\mathrm{b}}\:=x{\mathrm{E}}_{\mathrm{b}1}+(1-x){\mathrm{E}}_{\mathrm{b}2}\:$$

The equation of replication dynamics is shown in Eq. (4).

4 $$\begin{aligned}\:{{\mathrm{F}}_{\left( {\mathrm{x}} \right)}} & = {\mathrm{dx}}/{\mathrm{dt}} = x(1 - x)({{\mathrm{E}}_{{\mathrm{b}}1}} - {{\mathrm{E}}_{{\mathrm{b}}2}}) \\ & = x(x - 1)({R_B} - R_B^O + {C_B}y - R_B^Ny + R_B^Oy + K_B^OQ + K_B^NQy - \:K_B^OQy \\ & \quad - \:{C_B}\delta \:y - \alpha \:{S_G}z) \\ \end{aligned}$$

#### Platform organizer

The expected revenue,$\:{\mathrm{E}}_{\mathrm{S}1}$, can be derived from Eq. (5) when the platform organizer chooses to provide only the basic services.

5 $$\:{\mathrm{E}}_{\mathrm{S}1}=xz[{R}_{S}^{N}+(1-\alpha\:){S}_{G}-{C}_{S}^{N}+{I}_{S}-\delta\:{C}_{B}]+x(1-z)({R}_{S}^{N}-{C}_{S}^{N}+{I}_{S}-\:\:\:\:\:\:\:\delta\:{C}_{B})+(1-x)z[-{C}_{S}^{N}+{S}_{G}]+(1-x)(1-z)(-{C}_{S}^{N})\:$$

Otherwise, the expected revenue, $\:{\mathrm{E}}_{\mathrm{S}2}$, is specified in Eq. (2) when the platform organizer chooses to provide advanced services.

6 $$\:{\mathrm{E}}_{\mathrm{S}2}=xz({R}_{S}^{O}-{C}_{S}^{O})+x(1-z)({R}_{S}^{O}-{C}_{S}^{O})+(1-x)z(-{C}_{S}^{O})+(1-x)(1-\:\:\:\:\:\:\:z)(-{C}_{S}^{O})$$

Thus, the average return, denoted as$\:{\mathrm{E}}_{\mathrm{S}}$, is calculated as follows.

7 $$\:{\mathrm{E}}_{\mathrm{S}}=\mathrm{y}{\mathrm{E}}_{\mathrm{S}1}+(1-y){\mathrm{E}}_{\mathrm{S}2}\:\:\:$$

The equation of replication dynamics is shown in Eq. (8)

8 $$\:{\mathrm{F}}_{\left(\mathrm{y}\right)}=\mathrm{d}\mathrm{y}/\mathrm{d}\mathrm{t}=y(1-y)({\mathrm{E}}_{\mathrm{S}1}-{\mathrm{E}}_{\mathrm{S}2})\:\:\:\:\:\:\:\:\:\:\:\:=y(y-1)({C}_{S}^{N}-{C}_{S}^{O}-{I}_{S}x-{R}_{S}^{N}x+{R}_{S}^{O}x-{S}_{G}z+{C}_{B}\delta\:x+{S}_{G}\alpha\:xz)$$

#### Government

The expected return, $\:{\mathrm{E}}_{\mathrm{g}1}$, can be derived from Eq. (9) when the government chooses to offer subsidies.

9 $$\:{\mathrm{E}}_{\mathrm{g}1}=xy({R}_{G}^{N}-{C}_{G}-{S}_{G}+{F}_{S}+{F}_{B})+x(1-y)({R}_{G}^{O}-{C}_{G}-\alpha\:{S}_{G}+{F}_{B}+(1-\:\:\:\:\:\:\:x\left)y\right(-{C}_{G}-{S}_{G}+{F}_{S})+(1-x\left)\right(1-y\left)\right(-{C}_{G}\left)\right)$$

The expected return, $\:{\mathrm{E}}_{\mathrm{g}2}$ is specified in Eq. (10) when the government chooses not to offer subsidies.

10 $$\:{\mathrm{E}}_{\mathrm{g}2}=xy{R}_{G}^{N}+x(1-y){R}_{G}^{O}+(1-x)y+(1-x)(1-y)$$

The average expected return, denoted as$\:{\mathrm{E}}_{\mathrm{Z}}$, is calculated as follows.

11 $$\:{\mathrm{E}}_{\mathrm{Z}}=z{\mathrm{E}}_{\mathrm{g}1}+(1-z){\mathrm{E}}_{\mathrm{g}2}\:\:\:$$

The equation of replication dynamics is shown in Eq. (12).

12 $$\begin{aligned}\:{{\mathrm{F}}_{\left( {\mathrm{z}} \right)}} & = {\mathrm{dz}}/{\mathrm{dt}} = z(1 - z)({{\mathrm{E}}_{{\mathrm{g}}1}} - {{\mathrm{E}}_{{\mathrm{g}}2}}) \\ & = z(z - 1)({C_G} - {F_B}x - {F_S}y + {S_G}y + {S_G}\alpha \:x - {S_G}\alpha \:xy) \\ \end{aligned}$$

### Equilibrium stability analysis

In the dynamical system consisting of three subjects, setting the initial values as $\:{\mathrm{F}}_{\left(\mathrm{x}\right)}$=0,$\:{\:\mathrm{F}}_{\left(\mathrm{y}\right)}$= 0,$\:{\:\mathrm{F}}_{\left(\mathrm{z}\right)}$= 0 results in eight pure strategy equilibrium points for the system. These equilibrium points are: O(0, 0, 0), A(0, 0, 1), B(0, 1, 0), C(0, 1, 1), D(1, 0, 0), E(1, 0, 1), F(1, 1, 0), and G(1, 1, 1). We can compute the Jacobian matrix for the dynamic equations with these equilibrium points. According to evolutionary game theory, a model’s Evolutionarily Stable Strategy (ESS) is characterized by the condition that all eigenvalues of the Jacobi matrix evaluated at a particular point are negative. If all eigenvalues are positive, the point is unstable, while if the eigenvalues are both positive and negative, the point is a saddle point. Inserting the eight pure strategy equilibrium points into the system’s Jacobian matrix, we obtained the eigenvalues corresponding to each equilibrium point. Notably, the equilibrium point A(0, 0, 1) consistently yields a strictly positive eigenvalue $\:{\uplambda\:}3={\mathrm{C}}_{\mathrm{G}}>0$, and point B(0, 1, 0) also yields a strictly positive eigenvalue $\:{\uplambda\:}2={\mathrm{C}}_{\mathrm{S}}^{\mathrm{N}}\:-{\mathrm{C}}_{\mathrm{S}}^{\mathrm{O}}>0$. Because both points inherently possess at least one positive eigenvalue under any realistic parameter setting, they mathematically constitute unstable or saddle points. Consequently, these two scenarios can never evolve into an ESS and are therefore excluded from further detailed scenario analysis. By analyzing the signs of these eigenvalues, we determine whether each local equilibrium point qualifies as an ESS for the system, as summarized in Table 3.

Furthermore, we investigated the existence and stability of the interior mixed-strategy equilibrium point. By setting the replicator dynamic equations to zero $\:{\mathrm{F}}_{\left(\mathrm{x}\right)}$=0,$\:{\mathrm{F}}_{\left(\mathrm{y}\right)}$= 0,$\:{\mathrm{F}}_{\left(\mathrm{z}\right)}$= 0 while 0 < x, y, z < 1, the system may possess an interior equilibrium H(x, y, z). The existence of this mixed-strategy equilibrium is contingent upon the following system of equations:

13 $$\:{{\:\:\mathrm{R}}_{\mathrm{B}}-{\mathrm{R}}_{\mathrm{B}}^{\mathrm{O}}+\mathrm{C}}_{\mathrm{B}}\mathrm{y}-{\mathrm{R}}_{\mathrm{B}}^{\mathrm{N}}\mathrm{y}+{\mathrm{R}}_{\mathrm{B}}^{\mathrm{O}}\mathrm{y}+{\mathrm{K}}_{\mathrm{B}}^{\mathrm{O}}\mathrm{Q}+{\mathrm{K}}_{\mathrm{B}}^{\mathrm{N}}\mathrm{Q}\mathrm{y}-{\mathrm{K}}_{\mathrm{B}}^{\mathrm{O}}\mathrm{Q}\mathrm{y}-{\mathrm{C}}_{\mathrm{B}}{\updelta\:}\mathrm{y}-{\mathrm{S}}_{\mathrm{G}}\mathrm{a}\mathrm{z}=0$$

14 $$\:\:\:{\mathrm{C}}_{\mathrm{S}}^{\mathrm{N}}-{\mathrm{C}}_{\mathrm{S}}^{\mathrm{O}}-{\mathrm{I}}_{\mathrm{S}}\mathrm{x}-{\mathrm{R}}_{\mathrm{S}}^{\mathrm{N}}\mathrm{x}+{\mathrm{R}}_{\mathrm{S}}^{\mathrm{O}}\mathrm{x}-{\mathrm{S}}_{\mathrm{G}}\mathrm{z}+{\mathrm{C}}_{\mathrm{B}}{\updelta\:}\mathrm{x}+{\mathrm{S}}_{\mathrm{G}}\mathrm{a}\mathrm{x}\mathrm{z}=0$$

15 $$\:\:\:{\mathrm{C}}_{\mathrm{G}}-{\mathrm{F}}_{\mathrm{B}}\mathrm{x}-{\mathrm{F}}_{\mathrm{S}}\mathrm{y}+{\mathrm{S}}_{\mathrm{G}}\mathrm{y}+{\mathrm{S}}_{\mathrm{G}}\mathrm{a}\mathrm{x}-{\mathrm{S}}_{\mathrm{G}}\mathrm{a}\mathrm{x}\mathrm{y}=0$$

When the parameter settings ensure that the solutions satisfy 0 < x, y, z < 1, the interior equilibrium exists. However, regarding local stability, substituting H(x, y, z) into the Jacobian matrix shows that the trace and determinant cannot simultaneously satisfy the conditions for a stable sink. As mathematically proven in asymmetric multi-population evolutionary games^78,90^, such an interior mixed-strategy equilibrium is never asymptotically stable and strictly acts as a saddle point. Because boundedly rational subjects continuously adjust their strategies based on payoff differences, any slight perturbation will cause the system to diverge from H(x, y, z) and eventually converge to one of the pure-strategy boundaries. Therefore, the interior point does not constitute an ESS, and our focal analysis remains rigorously on the pure-strategy equilibria.

**Table 3:** Equilibrium stability analysis.

ESS	Stability conditions	Scenario number
O (0,0,0)	$\:{R}_{B}^{O}-{R}_{B}<{K}_{B}^{O}Q,\:\:{C}_{S}^{O}-{C}_{S}^{N}<0,\:-{C}_{G}<0$	1
C (0,1,1)	$\:{R}_{B}^{N}+{C}_{B}\delta\:+{S}_{G}\alpha\:-{C}_{B}-{K}_{B}^{N}Q<{R}_{B},\:{C}_{S}^{N}-{C}_{S}^{O}<{S}_{G},\:{C}_{G}+{S}_{G}<{F}_{S}$	2
D (1,0,0)	$\:{R}_{B}^{O}-{R}_{B}>{K}_{B}^{O}Q,\:\:{R}_{S}^{O}-{C}_{S}^{O}>{R}_{S}^{N}-{C}_{S}^{N}+{I}_{S}-{C}_{B}\delta\:,\:{C}_{G}+{S}_{G}\alpha\:>{F}_{B}$	3
E (1,0,1)	$\:(1-\delta\:){C}_{B}+{K}_{B}^{N}Q<{R}_{B}^{N}-{R}_{B},{R}_{S}^{O}-{C}_{S}^{O}<{R}_{S}^{N}-{C}_{S}^{N}+{I}_{S}-{C}_{B}\delta\:,$ $\:{F}_{B}+{F}_{S}<{S}_{G}+{C}_{G}$	4
F (1,1,0)	$\:{R}_{B}^{O}+{S}_{G}\alpha\:-{K}_{B}^{N}Q>{R}_{B},{R}_{S}^{O}-{C}_{S}^{O}>{R}_{S}^{N}-{C}_{S}^{N}+{I}_{S}+{C}_{B}\delta\:+(1-\alpha\:){S}_{G},$ $\:{F}_{B}>{C}_{G}+{S}_{G}\alpha\:$	5
G (1,1,1)	$\:{R}_{B}<{R}_{B}^{N}-(1-\delta\:){C}_{B}+{S}_{G}\alpha\:-{K}_{B}^{N}Q,{F}_{B}+{F}_{S}>{S}_{G}+{C}_{G},$ $\:{R}_{S}^{O}-{C}_{S}^{O}<{R}_{S}^{N}-{C}_{S}^{N}+(1-\alpha\:){S}_{G}-{C}_{B}\delta\:+{I}_{S}$	6

When conducting the numerical assessments, the additional expenditure incurred by the platform organizer to offer advanced services surpasses the cost of providing basic services as the default option ($\:{C}_{S}^{N}>{C}_{S}^{O}$). Furthermore, the advantage that a platform user enjoys from utilizing the advanced services after joining the platform is significantly higher than the advantage gained from using the basic services ($\:{R}_{B}^{N}>{R}_{B}^{O}$). Both of these advantages exceed the net benefit a user would receive if they did not join the platform at all. Additionally, when the government implements a subsidy strategy, the combined benefits for the users and the subsidies received exceed the total amount of government subsidies dispensed$\:\:{(F}_{B}+{F}_{S}>{S}_{G})$.

1. Scenario 1: Equilibrium point O (0,0,0).

In this scenario, the stable evolutionary strategy involves the platform organizer offering only basic services, users deciding not to join, and the government refraining from subsidizing the platform. Platform users are deterred from joining because the potential loss from using basic services outweighs the benefits. Meanwhile, without user participation, government subsidies, or additional revenue, the platform organizer finds that the cost of providing advanced services exceeds that of basic services alone, leading it to offer only basic services.

(2) Scenario 2: Equilibrium point C (0,1,1).

In this scenario, platform users’ decision to join or not hinges on the profits they gain from using advanced services. If this profit is less than the net profit derived from not joining, users are discouraged from participating. Conversely, users are motivated to join if the profit from using the services exceeds the profit from abstaining. Secondly, the platform organizer’s decision to continue offering advanced services depends on sufficient government subsidies to cover the associated costs. If this condition is met, the organizer will continue to provide these services. Finally, the government’s decision to persist in subsidy measures is determined by whether the social benefits gained outweigh the combined costs of formulating the policy and the total subsidy amount. Under these circumstances, the stable evolutionary strategy involves the organizer providing advanced services, users abstaining from joining, and the government continuing to offer subsidies.

(3) Scenario 3: Equilibrium point D (1,0,0).

In this scenario, when the incremental revenue garnered by platform users joining the platform and utilizing basic services outweighs their potential losses, and the net difference between the direct benefits of offering advanced services over basic services, coupled with the cumulative indirect benefits, is sufficient to balance the disparity in costs between them and the collective cost sharing for platform users utilizing advanced services, it becomes advantageous. Furthermore, if the realized benefits exceed the management expenses associated with subsidies and the total subsidy amount, it becomes a viable strategy for all stakeholders. Under these conditions, the stable approach for all parties would involve users opting to join the platform, the organizer committing to providing basic services, and the government abstaining from providing subsidies.

Scenario 4: Equilibrium point E (1,1,0).

In this scenario, when the platform organizer shares some of the costs of advanced services with users and the benefits users gain from these services outweigh potential losses, users are likely to actively choose them. As a result, the platform organizer profits more from offering advanced services than from providing only basic ones. However, for the government, the total subsidy amount and administrative costs associated with providing subsidies exceed the reputation benefits gained. Hence, a rational government would refrain from offering subsidies. Given these considerations, the stable evolutionary strategy for all parties involved would be for the platform organizer to provide advanced services, for platform users to join the platform, and for the government to refrain from offering subsidies.

Scenario 5: Equilibrium point F (1,0,1).

In this scenario, when the benefits of subsidizing outweigh the total subsidy and management costs, the government adopts subsidy measures to encourage users to join the platform. With these subsidies, users find it more profitable to use the platform’s basic services. Meanwhile, the platform organizer realizes that the profit margin from providing basic functions exceeds that from advanced functions, so it offers only the former. Consequently, the stable strategy for all parties involved is that users join the platform, the organizer provides basic functions, and the government offers subsidies.

Scenario 6: Equilibrium point G (1,1,1).

In this scenario, platform users find that the benefits of joining the platform and using advanced functions, subsidized by the government and cost-shared by the platform organizer, outweigh the benefits of not joining. Therefore, users choose to join the platform. The platform organizer also provides advanced functions, as the direct profit exceeds the cost of basic services. The government implements subsidies as the total social benefits exceed the costs. This mutually beneficial strategy enables all parties to effectively use resources and respond positively to one another’s actions.

## Simulation analyses

In the nascent stages of the platform’s evolution, the government introduces subsidies to motivate the platform organizer to offer advanced services, despite initial user reluctance due to potential losses and the high cost of services, represented by C (0,1,1). To foster the development of the emerging ISECO, the platform organizer implements a cost-sharing approach with users, leveraging government subsidies to ensure that users derive greater benefits from utilizing the advanced services than from not participating. This propels the platform towards a state of G (1,1,1). As the platform matures, cooperation stabilizes, risks diminish, and benefits expand. Simultaneously, the organizer’s cost of providing advanced services decreases due to innovations and technological advancements, enabling the government to gradually phase out subsidies and ultimately reach a subsidy-free status of E (1,1,0). To decipher the pivotal factors driving the transformation from a software provider to a platform organizer, this process is segmented into three distinct stages, each undergoing rigorous analysis and simulation using MATLAB R2018a to delve into the dynamics and influencing factors of this critical transition.

### Parameter settings

In this section, we perform numerical simulation experiments. To guarantee the wide applicability and relevance of the derived patterns, the numerical settings adhere to the following principles: Firstly, the numerical disparities between variables are kept realistic. Secondly, the values of each equilibrium point are calibrated based on the theoretical analysis presented in Sect. 4. Lastly, whenever equilibrium points shift, changes in variable values are kept minimal to prevent disproportionate quantities, thereby facilitating a subsequent in-depth sensitivity analysis of the parameters^91,92^. The initial numerical configurations are outlined in Table 4.

**Table 4:** Parameter configuration.

Parameter	Parameter value		
	Initial stage	Maturity stage	Ideal stage
$\:\alpha\:$	0.2	0.4	0.4
$\:\delta\:$	0.1	0.2	0.2
$\:{R}_{S}^{N}$	75	75	78
$\:{R}_{S}^{O}$	72	72	72
$\:{R}_{B}^{N}$	29	29	32
$\:{R}_{B}^{O}$	28	28	28
$\:{C}_{B}$	13	13	13
$\:{K}_{B}^{N}$	0.35	0.35	0.15
$\:{K}_{B}^{O}$	0.05	0.05	0.05
*Q*	15	15	15
$\:{S}_{G}$	15	25	30
$\:{R}_{B}$	18	18	18
$\:{C}_{S}^{N}$	40	40	35
$\:{I}_{S}$	2	2	2
$\:{C}_{S}^{O}$	30	30	30
$\:{C}_{G}$	5.5	5.5	5.5
$\:{F}_{B}$	30	30	10
$\:{F}_{S}$	30	30	10

To ensure the simulation is grounded in real-world economic logic, baseline parameters were calibrated using CAXA’s strategic transition. Our parameters reflect relative financial ratios and industry norms.

Platform development entails significantly higher capital expenditure than traditional software maintenance ($\:{\mathrm{C}}_{\mathrm{S}}^{\mathrm{N}}>{\mathrm{C}}_{\mathrm{S}}^{\mathrm{O}}$). Consequently, the initial organic profit of the platform often lags behind legacy systems ($\:{\mathrm{R}}_{\mathrm{S}}^{\mathrm{N}}-{\mathrm{C}}_{\mathrm{S}}^{\mathrm{N}}<{\mathrm{R}}_{\mathrm{S}}^{\mathrm{O}}-{\mathrm{C}}_{\mathrm{S}}^{\mathrm{O}}$), mathematically justifying the necessity of external incentives. Subsidies are calibrated to offset approximately 20% to 30% of the platform’s R&D costs, in line with China’s funding policies for Specialized and Innovative manufacturing enterprises.

Furthermore, user heterogeneity is inherently captured by dynamically varying the initial probability states, which represent the diverse initial willingness of different stakeholder cohorts. As demonstrated in our subsequent sensitivity analyses, the system exhibits robust stability. Provided the parameters remain within the topological thresholds derived from the Jacobian matrix, the equilibria successfully resist minor parameter perturbations and heterogeneous initial states, consistently converging to the predicted Evolutionarily Stable Strategy (ESS).

### Simulation results

1. The initial stage.

**Fig. 4:**
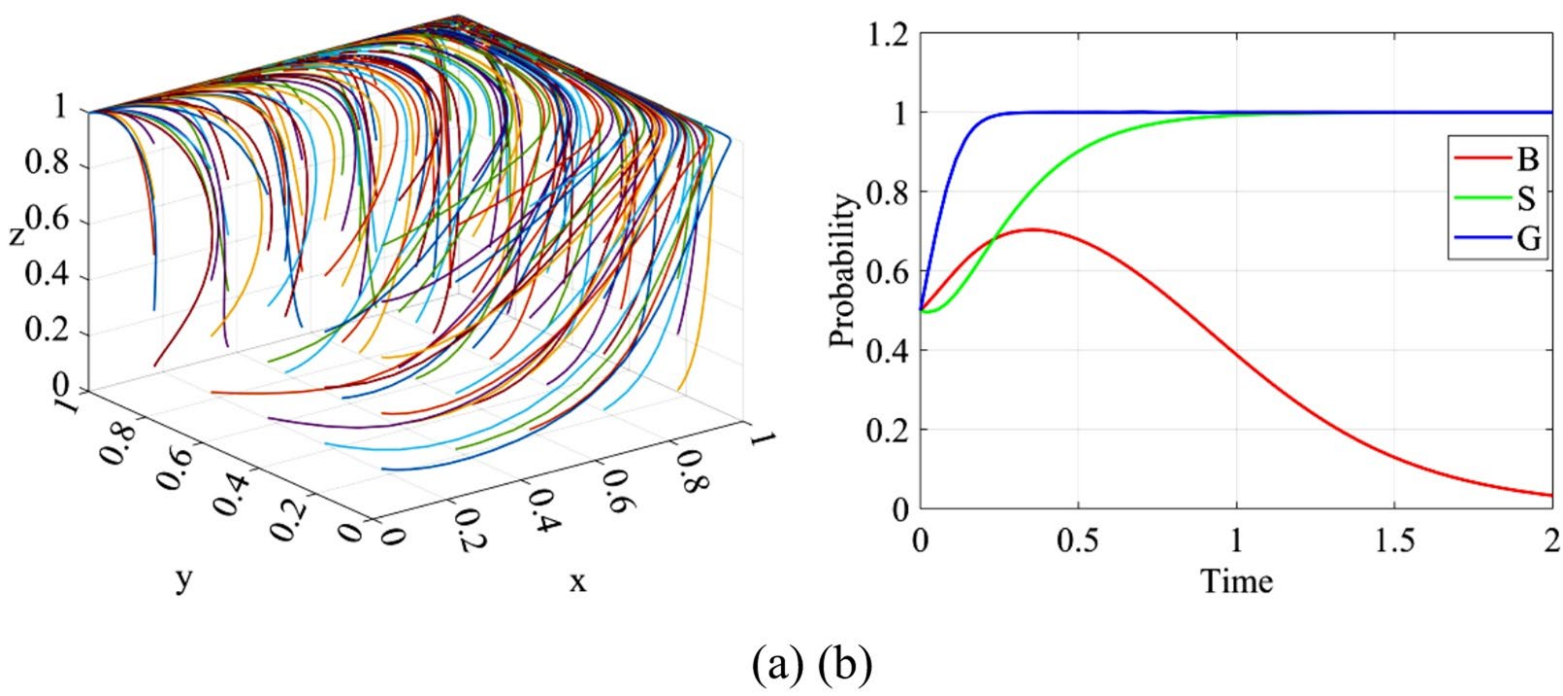
Evolutionary trends in the initial stage.

Figure 4 illustrates the evolutionary trajectories of the platform users, the organizer, and the government over 500 iterations, shown in three-dimensional (a) and two-dimensional (b) views. The figures reveal that, despite varying initial strategies, all parties ultimately converge on the point (0, 1, 1). This convergence signifies that the government adopts subsidy policies while the platform organizer introduces advanced functions. However, platform users do not engage with the platform. This result supports our earlier analysis, demonstrating that government subsidies and the platform’s cost-sharing mechanisms significantly impact users’ strategic decisions during the platform’s early development stages.

(2) The mature stage.

**Fig. 5:**
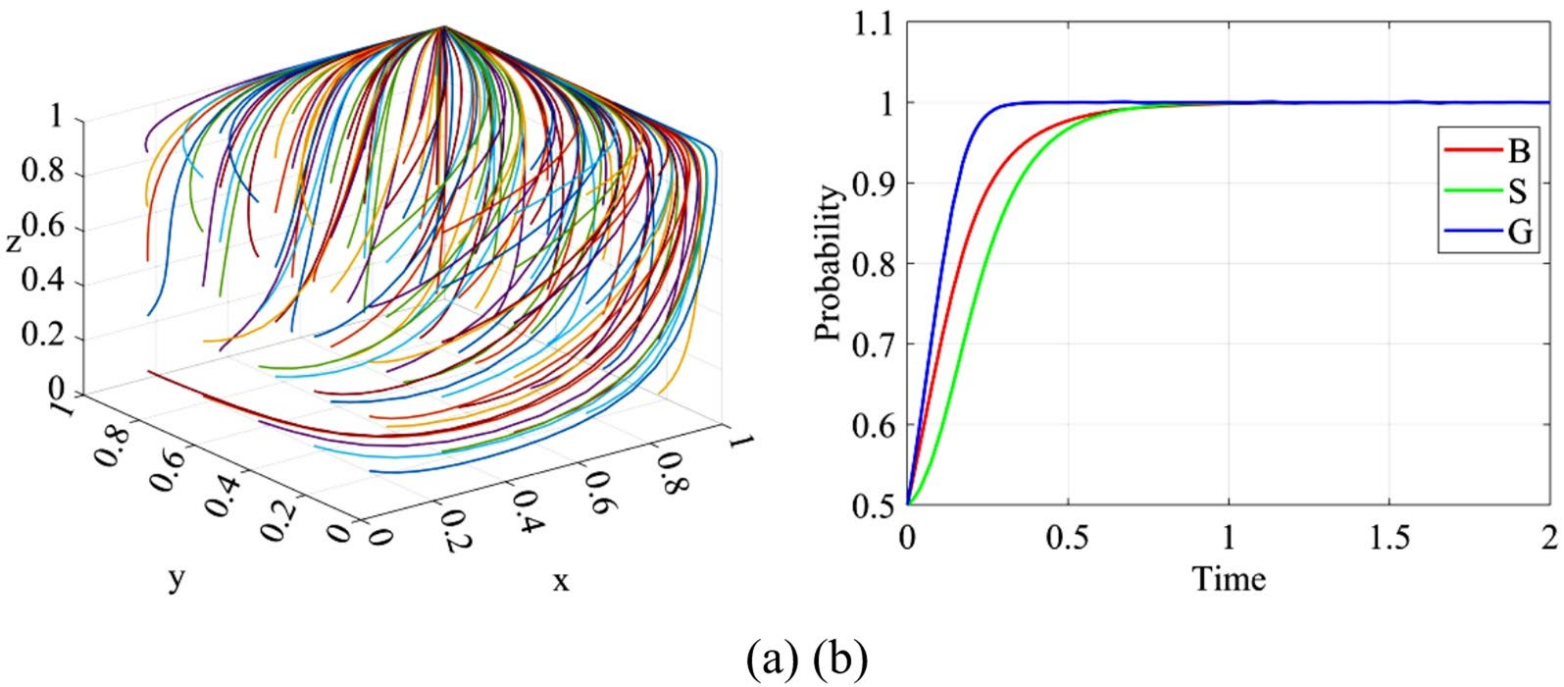
Evolutionary trends in the maturity stage.

Figure 5 illustrates that all parties converge on the stable strategy (1,1,1) in both the three-dimensional (a) and two-dimensional (b) displays: users joining the platform, the organizer offering advanced features, and the government providing subsidies. This trend highlights that users are more likely to join the platform as subsidies and shared costs increase.

(3) The ideal stage.

**Fig. 6:**
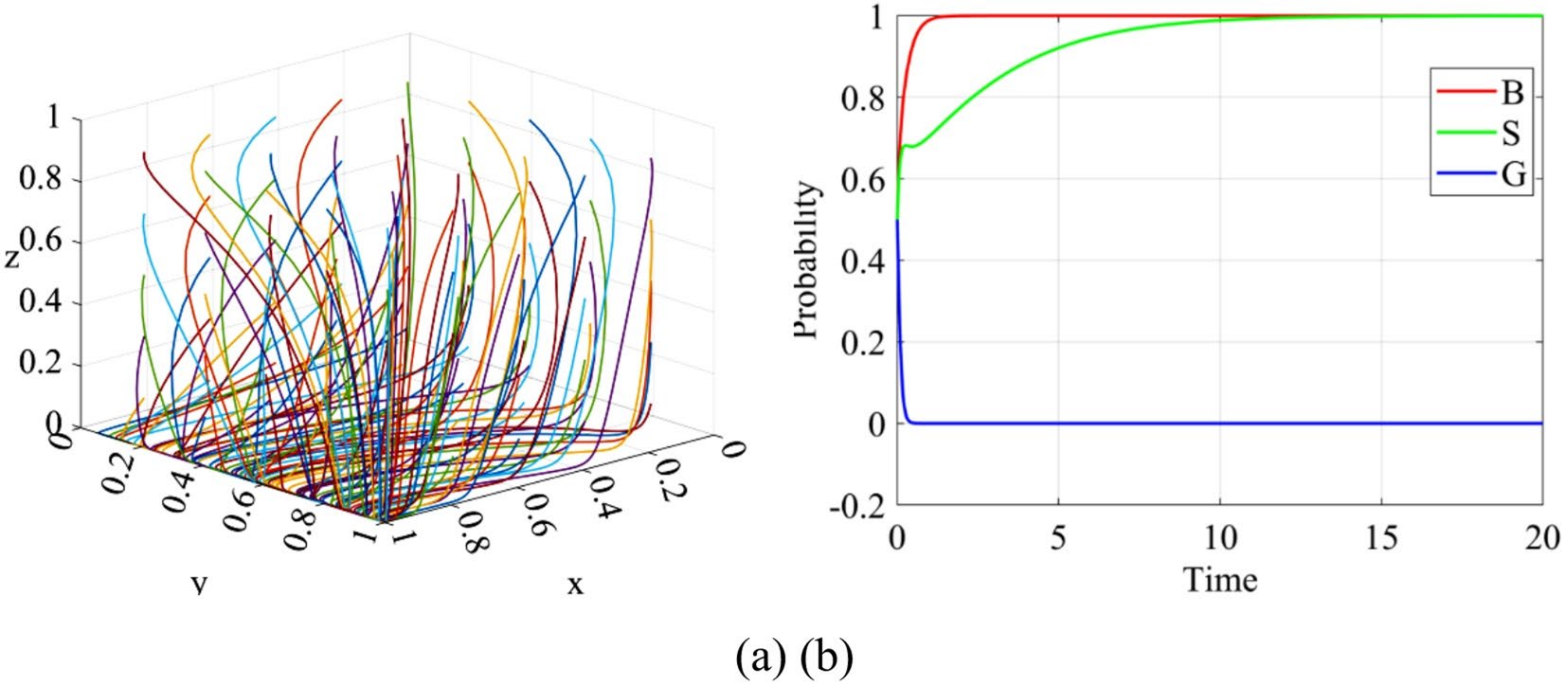
Evolutionary trends in the ideal stage.

In the ideal state, simulations indicate convergence to the strategy (1,1,0), representing a unique Evolutionary Stable Strategy (ESS). Figure 6 depicts the system’s evolutionary trajectories in three-dimensional (a) and two-dimensional (b) views. It shows that the platform users and the organizer benefit from advanced features. As the government’s gains from providing subsidies decrease, it gradually reduces its market intervention.

### Key coefficient analyses

#### Government subsidy allocation coefficient

**Fig. 7:**
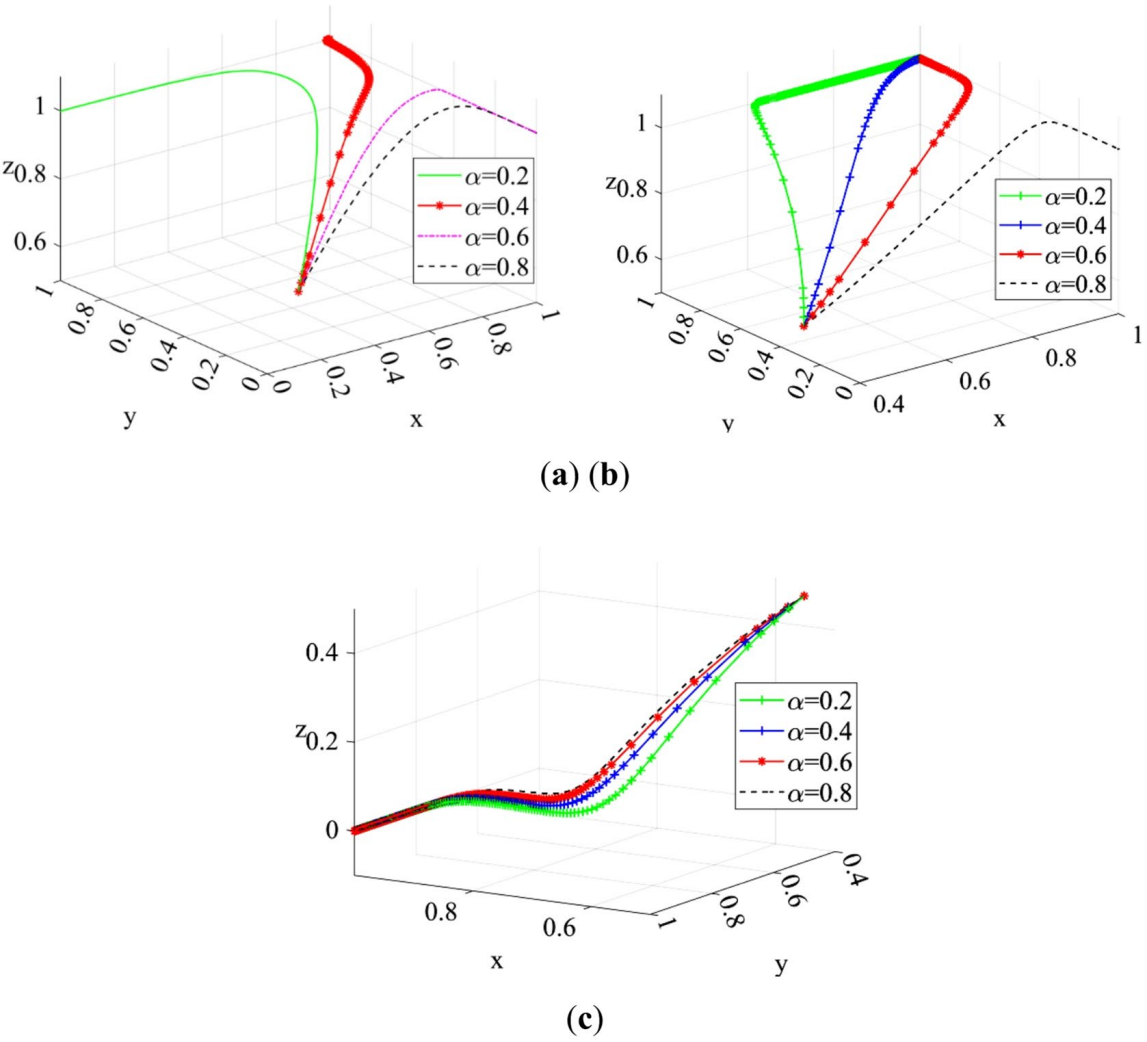
The effect of $\:\alpha\:$ on the evolutionary stability of the ISECO.

**Fig. 8:**
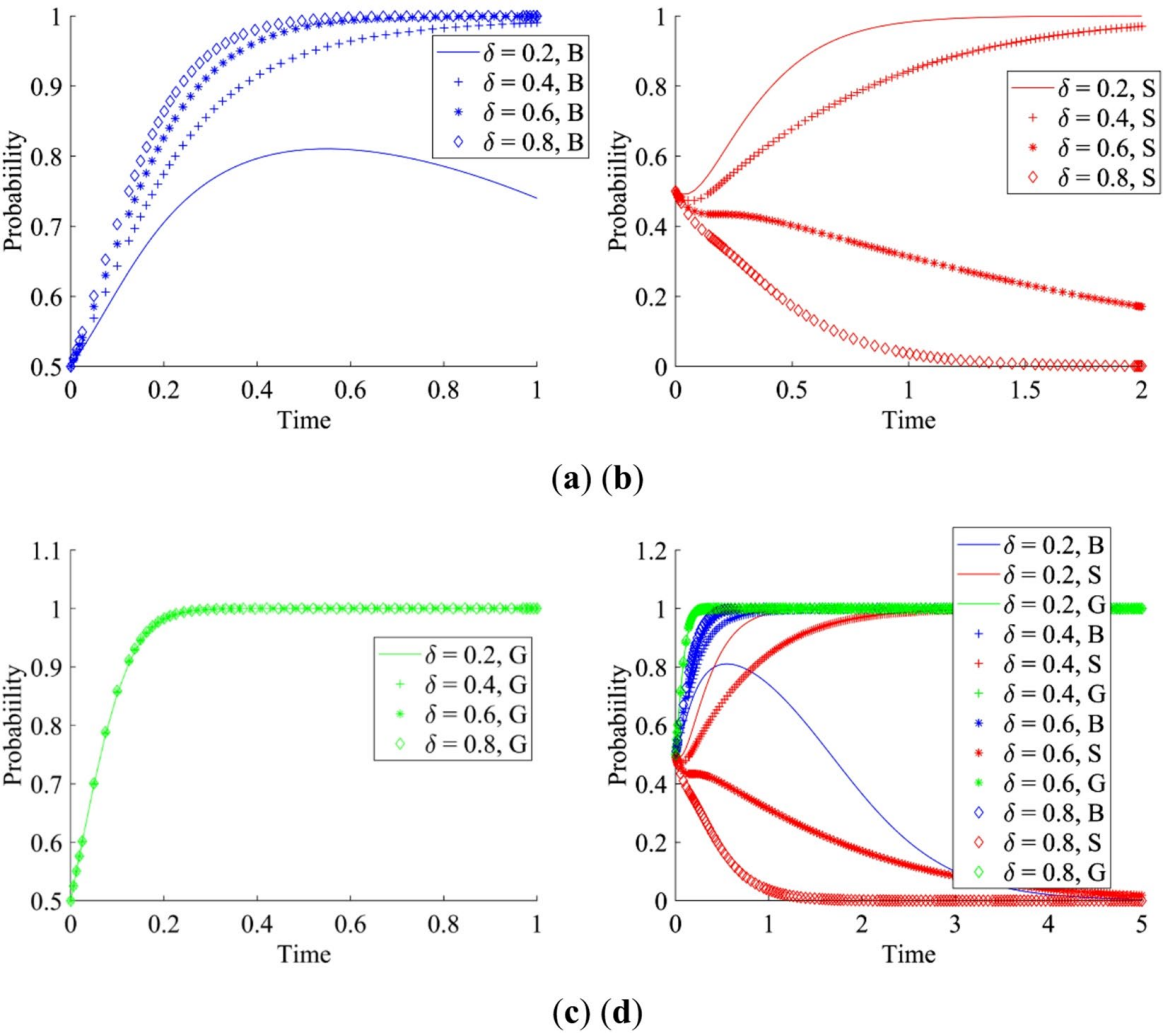
The sensitivity analysis of $\:\delta\:$ on the platform users (**a**), the organizer (**b**), the government (**c**), and the ISECO (**d**).

We analyze the impact of the government subsidy allocation coefficient $\:\alpha\:$ on the evolutionary stability of the ISECO by simulating different parameter values (0.2, 0.4, 0.6, 0.8). Figure 7 shows that this coefficient $\:\alpha\:$ has a significant influence in the initial stage (a), the mature stage(b), and the ideal stage (c). A subsidy allocation below 0.4 discourages platform users from participating, while one above 0.4 substantially incentivizes their joining. For platform organizers, a coefficient below 0.6 prompts them to offer advanced functions, whereas a coefficient above 0.6 prompts them to offer only basic functions. However, in the ideal scenario presented in Fig. 8c, the coefficient does not determine strategy selection but rather affects the speed at which the system reaches its evolutionary stable state. Specifically, a higher proportion of subsidies allocated to users accelerates the evolution towards the (1,1,0) stable point.

in initial (a), mature (b), and ideal (c) stages.

#### Cost-sharing ratio

**Fig. 9:**
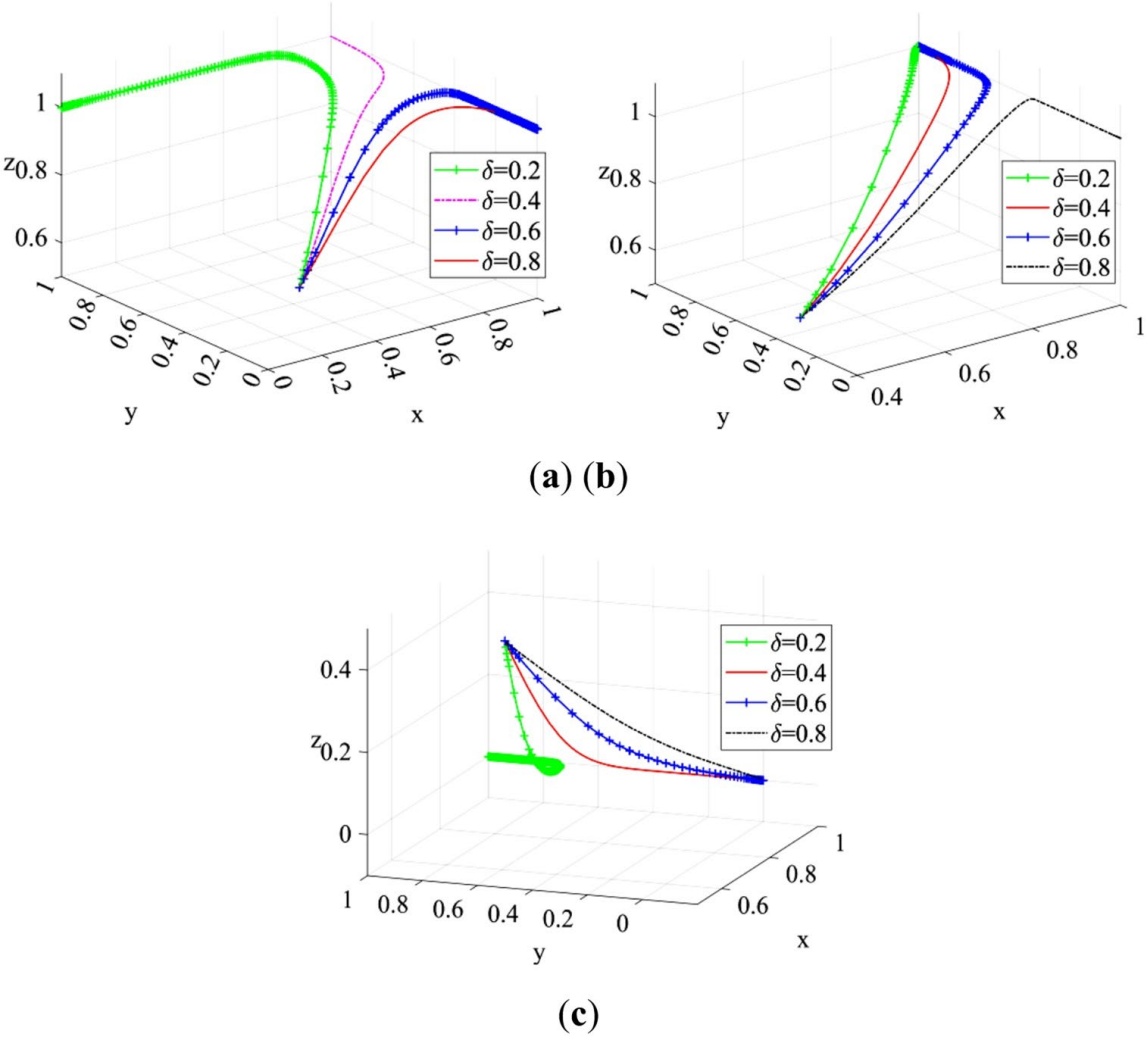
The effect of $\:\delta\:$ on the evolutionary stability of the ISECO.

Through simulation, we examine the effects of varying cost-sharing ratios. $\:\delta\:$ on the evolutionary stability of the ISECO system by setting $\:\delta\:$ to 0.2, 0.4, 0.6, and 0.8, as presented in Fig. 9. In the initial stage, we identify two critical ranges for $\:\delta\:$. When $\:\delta\:$ Falls between 0.2 and 0.4. Users are motivated to join because of reduced costs for advanced features, which stabilize at the equilibrium point (1,1,1). However, when $\:\delta\:$ ranges from 0.4 to 0.6, the increased costs for the platform organizer prompt them to offer only basic functions, resulting in stability at (1,0,1). In the maturity stage, the system stabilizes at (1,1,1) for $\:\delta\:$ below a certain threshold and shifts to (1,0,1) when $\:\delta\:$ exceeds this threshold. Finally, in the ideal state, the critical range for $\:\delta\:$ is again between 0.2 and 0.4. Below this range, the system stabilizes at (1,1,0); however, when $\:\delta\:$ exceeds 0.4, it stabilizes at (1,0,0).

in initial (a), mature (b), and ideal (c) stages.

### Sensitivity analysis

#### Government subsidy coefficient

**Fig. 10:**
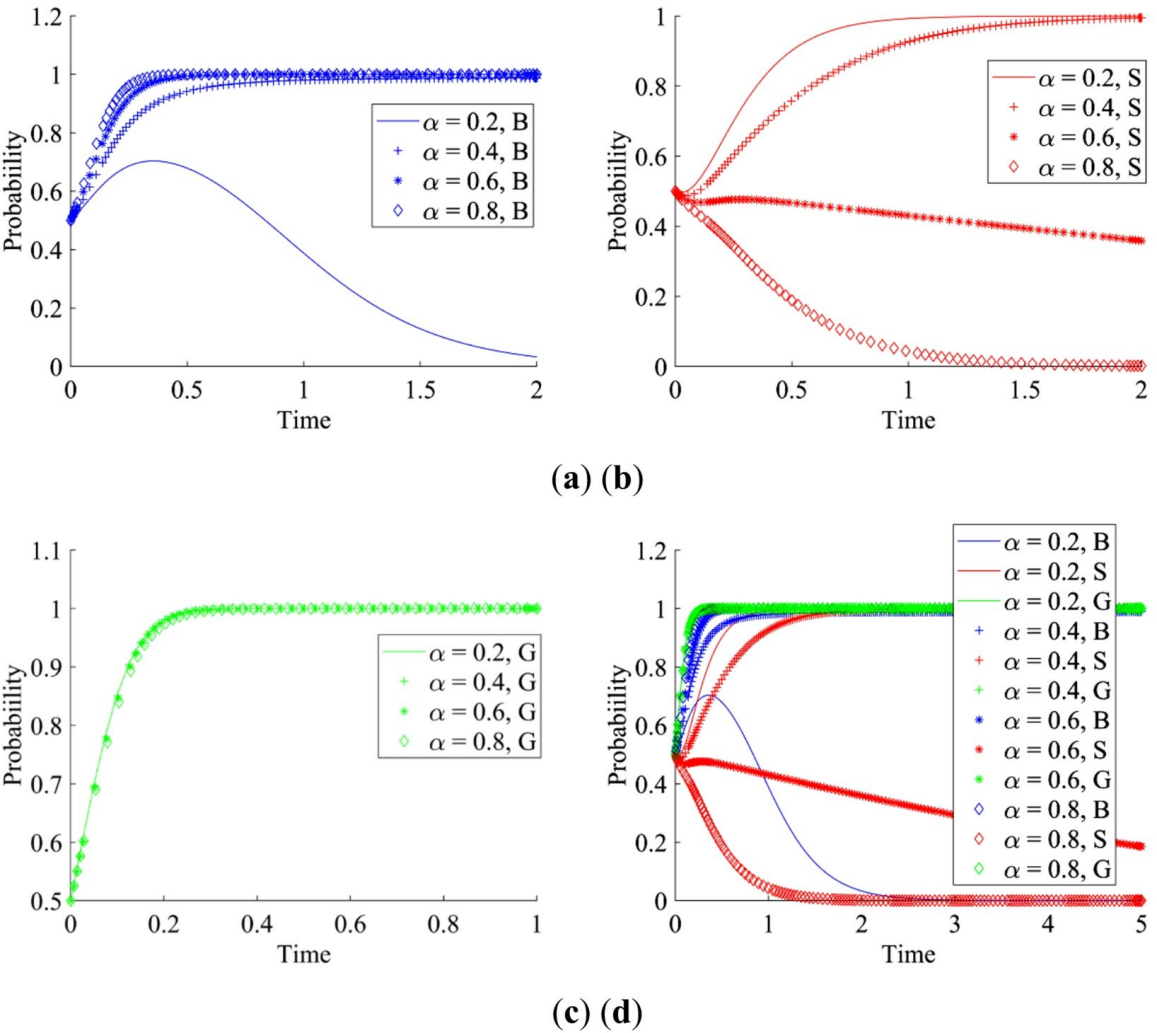
The sensitivity analysis of $\:\alpha\:$ on the platform users (**a**), the organizer (**b**), the government (**c**), and the ISECO (**d**).

The government subsidy coefficient $\:\alpha\:$ plays a pivotal role in shaping the strategic choices of both the platform organizer and users. As depicted in Fig. 10, the government’s subsidy allocation at varying levels significantly affects the evolution and stability of the ISECO. The key threshold for users lies in the subsidy coefficient range of 0.2 to 0.4, which determines whether to join the platform. Conversely, for the platform organizer, the critical threshold falls within a subsidy coefficient range of 0.4 to 0.8, dictating whether offering advanced services is financially viable. If the subsidy coefficient exceeds this range, the organizer may shift to providing basic services due to reduced profitability. Thus, the subsidy coefficient has significant implications for ecosystem stability.

#### Cost-sharing ratio

The cost-sharing ratio $\:\delta\:$ also plays a crucial role in shaping the strategic choices of the platform organizer and users. Figure 8 shows a threshold for platform users of 0.2–0.4. Specifically, users are willing to participate on the platform when the cost-sharing ratio falls within this range. On the other hand, for the platform organizer, a different threshold exists in the range of 0.4 to 0.8. This range is critical in determining whether offering advanced services remains financially viable. Should the cost-sharing ratio exceed this threshold, profitability considerations may lead the organizer to pivot toward providing basic services only.

## Discussion

### The necessity of government interventions

This study introduces a novel perspective on business ecosystems by applying evolutionary game theory, enabling a more dynamic and nuanced analysis than prior research. Prior studies predominantly employed system dynamics or agent-based models, which emphasize macro-level changes or individual behaviors but face limitations in addressing the long-term evolution of strategies and the stability of ecosystems^72,75^. In contrast, this study employs an evolutionary game model to underscore the critical role of factors such as government subsidies and cost-sharing strategies in enabling traditional software suppliers to transform into ecosystem leaders. The findings reveal that these policies and strategies exert a substantial influence on the stability and dynamics of the ecosystem throughout its evolutionary trajectory. By enhancing the theoretical framework, this research provides practical insights for policymakers and strategic planners.

This study extends the existing literature on industrial software ecosystem evolution by incorporating an evolutionary game-theoretic approach to model the transition from traditional software suppliers to platform organizers. Compared with prior research that primarily relies on system dynamics^75^ or agent-based models^72^, our study highlights the dynamic strategic interactions among key stakeholders and uncovers distinct evolutionary pathways.

One key contribution of our findings is the identification of government subsidies and cost-sharing mechanisms as critical determinants in shaping the evolutionary trajectory of industrial software ecosystems. Prior studies^24,76^ have also emphasized the role of policy interventions; however, our results offer a more nuanced perspective by illustrating how these mechanisms shape distinct equilibrium states. Specifically, our simulation results indicate that government subsidies play a pivotal role in the early stage of platform formation by reducing user participation costs and incentivizing software firms to offer advanced services. These dynamic results confirm that during the process of software vendors’ transition to the platform model, the initial platform architecture costs in the early stage far exceed the marginal ecosystem benefits. Our model mathematically verifies that without precise external incentives, an organic, market-driven transformation is prone to failure at this stage. This finding is consistent with previous research on platform emergence^42^, which highlights the importance of external incentives in fostering ecosystem development. Consequently, our findings carry important policy implications: the role of government intervention is crucial during the early stages of ecosystem formation. Targeted subsidies can facilitate user adoption and encourage software providers to transition towards a platform model. However, these subsidies should not be indefinite; rather, they should be gradually phased out as the ecosystem matures, ensuring a smooth transition to a self-sustaining model. Policymakers should design dynamic subsidy policies that respond to changes in ecosystem structure, rather than adopting static, one-size-fits-all approaches.

### Cost-sharing mechanisms and policy thresholds

Moreover, our study offers an alternative explanation for the self-sustaining nature of mature software ecosystems. While earlier studies have attributed ecosystem stability to network effects and technological modularity^10,25^, our model suggests that strategic cost-sharing arrangements between platform organizers and users serve as an equally important mechanism. The transition from a government-supported phase to a market-driven phase—characterized by the gradual withdrawal of subsidies—is an important insight that aligns with the broader literature on digital platform governance^5,54^. These results suggest that policymakers should adopt a phased approach to subsidies, ensuring that platforms develop endogenous mechanisms for long-term sustainability.

Another notable finding is the highly policy-relevant threshold effect associated with cost-sharing ratios and subsidy allocation. Our sensitivity analysis transforms mathematical parameters into concrete policy boundaries. On the lower end, subsidies must exceed a specific activation threshold to overcome initial sunk costs; otherwise, policy efforts become ineffective and scattered investments. Conversely, beyond a certain upper threshold, excessive cost-sharing burdens on platform organizers may discourage them from offering advanced services, potentially undermining the entire ecosystem. Furthermore, exceeding subsidy ceilings without a planned withdrawal mechanism risks inducing moral hazard and over-reliance among enterprises. This insight extends prior work on cost-benefit trade-offs in business ecosystems^80^. It suggests that firms should adopt a balanced strategy that optimizes user incentives without compromising their own profitability. From a managerial perspective, an optimal cost-sharing strategy is essential for platform organizers to maintain ecosystem sustainability. While subsidizing user costs can accelerate platform adoption, excessive cost-sharing may strain finances and reduce incentives to offer advanced services. Firms should continuously monitor the cost-benefit dynamics and adjust their strategies to maintain a balance between user incentives and platform profitability.

### Evolutionary pathways and model generalizability

**Fig. 11:**
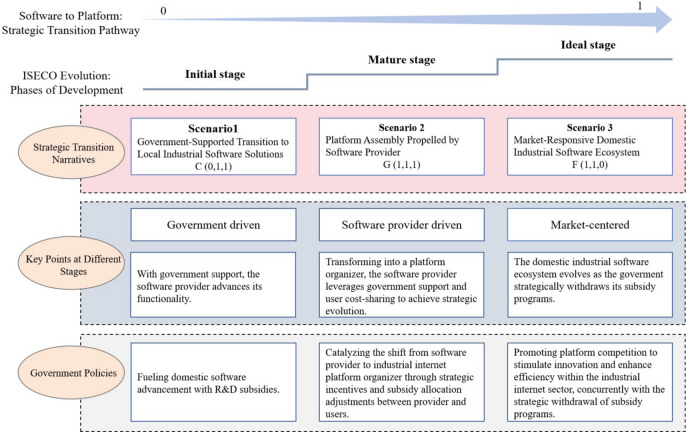
The strategic transition from a software provider to a platform organizer.

The evolutionary game theory model presented in this study elucidates the transformation process of software companies into ecosystem leaders across three distinct stages: initial, mature, and ideal, as depicted in Fig. 11. The initial stage is characterized by stable operations, supported by government subsidies and represented by state (0,1,1), wherein the company has a strong product foundation but a limited user base. As the company strategically invests and collaborates, it integrates into the ecosystem, transitioning to state (1,1,1), signifying an expanded user base and its emergence as an ecosystem leader. In the ideal stage, the company optimizes its role within the ecosystem, achieving state (1,1,0), at which point government subsidies are withdrawn, marking the realization of a self-sustaining domestic software ecosystem. The simulation results underscore the profound impact of government subsidies and cost-sharing strategies on this evolutionary process, emphasizing their importance for policymakers and industry stakeholders in fostering ecosystem development. The model’s insights affirm the pivotal role of factors such as government subsidy allocation and platform cost-sharing ratios in steering the system towards an Evolutionary Stable Strategy (ESS), while revealing threshold effects that shape strategic decisions and ecosystem stability.

Finally, regarding the general applicability of our method, although our three-role model adopts a binary strategy-selection approach, this abstraction closely reflects the true state of real-world ecosystems. Although daily operational strategies are continuous, macro-level strategic leaps, such as a traditional software provider deciding whether to invest heavily in migrating to a cloud platform, are essentially discrete, either-or decisions at the board level. By capturing these core structural tensions and binary strategy intersections, our model can filter out micro-level disturbances and provide a universal framework for understanding the emergence and governance of platforms across different industrial fields.

Building upon these insights, the findings highlight the importance of stakeholder coordination in ecosystem development. Governments, platform organizers, and users must engage in a collaborative process to dynamically adjust policies and strategies to align with evolving market conditions. This insight is particularly relevant for emerging economies, where industrial software platforms are still in their nascent stages and require concerted efforts from multiple actors to establish a robust ecosystem. Ultimately, from a theoretical perspective, this study contributes to the understanding of Industrial Software Ecosystems (ISECO) by integrating evolutionary game theory to analyze the strategic interactions among stakeholders. In practice, the findings offer valuable insights for policymakers aiming to foster the growth of the domestic software industry, while serving as a decision-support tool to help platform organizers and users understand the implications of strategic choices, thereby enhancing decision-making processes.

## Conclusions and implications

This study establishes that a critical sequential dependency governs the strategic transition from traditional software providers to platform organizers. Specifically, overcoming the early-stage financial deficit of platform architecture strictly requires external government subsidies to initiate ecosystem emergence. However, achieving long-term, self-sustaining ecosystem stability fundamentally depends on a timely strategic shift from reliance on external policies to an endogenous, market-driven cost-sharing mechanism. Furthermore, the evolution of industrial software ecosystems follows a structural trajectory comprising three stages: the initial, mature, and ideal stages. The dynamic threshold boundaries of these intervention measures determine the form of these ecosystems.

Theoretically, this research operationalizes the thresholds required for ecosystem stability. Practically, it provides a macro-level decision-support framework, asserting that policymakers must adopt phased subsidy withdrawals to prevent moral hazard. At the same time, platform organizers must dynamically balance user cost-sharing ratios to prevent ecosystem collapse.

Despite these contributions, several limitations warrant further exploration. First, the EGT framework relies on bounded rationality, which captures gradual strategic adjustments but may overlook sudden, irrational market shifts. Second, the simplified assumption of binary strategy choices, while necessary for deriving clear macro-level equilibria, cannot fully encompass the complexity of real-world enterprise decision-making. Finally, the derived equilibria depend fundamentally on the defined payoff structure; extreme parameter variations could alter the evolutionary trajectories. Furthermore, the simulation relies on short-term data, whereas real-world transformations involve broader influencing factors. Future research can address these boundaries by integrating long-term empirical microdata, exploring continuous strategy spaces, or combining EGT with more refined behavioral models and multiple case studies to provide a more comprehensive understanding of platform evolution.

## Data Availability

Our simulation codes and underlying data are available at the following DOI: https://doi.org/10.5281/zenodo.19754345.
